# Neuraminidase-1 promotes heart failure after ischemia/reperfusion injury by affecting cardiomyocytes and invading monocytes/macrophages

**DOI:** 10.1007/s00395-020-00821-z

**Published:** 2020-09-25

**Authors:** Maren Heimerl, Irina Sieve, Melanie Ricke-Hoch, Sergej Erschow, Karin Battmer, Michaela Scherr, Denise Hilfiker-Kleiner

**Affiliations:** 1 Molecular Cardiology, Department of Cardiology and Angiology, Hannover Medical School, Carl-Neuberg Str. 1, 30625 Hannover, Germany; 2 Department of Hematology, Hemostasis, Oncology and Stem Cell Transplantation, Hannover Medical School, Carl-Neuberg-Str.1, 30625 Hannover, Germany

**Keywords:** Neuraminidase 1, Sialidase 1, Ischemia/reperfusion, Inflammation, Monocytes

## Abstract

Neuraminidase (NEU)1 forms a multienzyme complex with beta-galactosidase (β-GAL) and protective-protein/cathepsin (PPC) A, which cleaves sialic-acids from cell surface glycoconjugates. We investigated the role of NEU1 in the myocardium after ischemia/reperfusion (I/R). Three days after inducing I/R, left ventricles (LV) of male mice (3 months-old) displayed upregulated neuraminidase activity and increased NEU1, β-GAL and PPCA expression. Mice hypomorphic for *neu1* (hNEU1) had less neuraminidase activity, fewer pro-inflammatory (Lin^−^CD11b^+^F4/80^+^Ly-6C^high^), and more anti-inflammatory macrophages (Lin^−^CD11b^+^F4/80^+^Ly-6C^low^) 3 days after I/R, and less LV dysfunction 14 days after I/R. WT mice transplanted with hNEU1-bone marrow (BM) and hNEU1 mice with WT-BM showed significantly better LV function 14 days after I/R compared with WT mice with WT-BM. Mice with a cardiomyocyte-specific NEU1 overexpression displayed no difference in inflammation 3 days after I/R, but showed increased cardiomyocyte hypertrophy, reduced expression and mislocalization of Connexin-43 in gap junctions, and LV dysfunction despite a similar infarct scar size to WT mice 14 days after I/R. The upregulation of NEU1 after I/R contributes to heart failure by promoting inflammation in invading monocytes/macrophages, enhancing cardiomyocyte hypertrophy, and impairing gap junction function, suggesting that systemic NEU1 inhibition may reduce heart failure after I/R.

**Electronic supplementary material:**

The online version of this article (10.1007/s00395-020-00821-z) contains supplementary material, which is available to authorized users.

## Introduction

Despite the successful implementation of reperfusion strategies to rescue the ischemic myocardium and thereby to reduce acute death, the long-term morbidity and mortality of patients with heart failure, due to adverse remodelling, have increased. Inflammation triggers not only the ischemic insult but also the reperfusion injury is considered to modulate post-MI outcome [17].Cell-to-cell communication plays an important role in cardiac performance after an ischemic insult. In this regard, the glycocalyx, composed of glycoconjugates and proteoglycans, is a key element for inter- and intracellular communication and tissue homeostasis [37]. Important components of the glycocalyx are sialic aids, 9-C-backbone sugars, which are bound terminally to glycoproteins and glycolipids on the plasma membrane of vertebrate and higher invertebrate cells [8]. By bearing a carboxyl group, they contribute to the total negative charge of the outer plasma membrane and thus affect not only protein–protein interactions by steric hindrance but also cell–cell and cell–matrix interactions [36, 37, 41]. In pathophysiological conditions (atherosclerosis, infection, ischemic injury, diabetes, trauma, and acute lung injury), the sialic acid content is altered by sialidases (also called neuraminidases, NEU) [37]. In addition, increased neuraminidase activity has been described in the plasma of patients after myocardial infarction (MI) compared with healthy controls [15]. For cardiomyocytes, it has been reported that an aberrant sialylation or desialylation of the plasma membrane results in disturbed functionality of cardiac ion channels, which may lead to arrhythmias [3, 11, 29].

In the mammalian system, four NEU enzymes (NEU1-4) are expressed in a cell and development-specific manner [37]. Of these, NEU1 has initially been described as a lysosomal protein which plays a role in the catabolism of glycosylated proteins [35]. NEU1 can self-associate, but for catalytical activation and protection from lysosomal degradation, it needs to form a multienzyme complex with β-GAL and protective PPCA [4]. Chronically reduced NEU1 activity leads to various pathologies including lysosomal storage disorder sialidosis, autoimmune diseases, and the malignancy and metastasis of cancer cells [32]. We previously showed that enhanced NEU1 expression promotes an inflammatory phenotype in monocytes/macrophages, as indicated by higher expression of pro-inflammatory cytokines and higher phagocytic activity with a direct positive feedback loop with interleukin (IL)-1β [37]. Moreover, we showed that monocytes/macrophages which highly express NEU1 are located within human atherosclerotic plaques and lesions, and circulating monocytes from MI patients showed elevated NEU1 mRNA expression compared with healthy controls [37]. With regard to the heart, inflammation has been identified as a crucial element for cardiac remodelling and outcome after I/R injury [14, 16, 20, 40]. Hereby, the different populations of monocytes/macrophages with partly opposing features orchestrate and determine healing but also detrimental remodelling [21, 30, 40]. Importantly, at an early stage after the ischemic insult, a predominance of pro-inflammatory leukocyte antigen 6C-(Ly-6C^high^) bearing monocytes/macrophages produce pro-inflammatory cytokines and digest infarcted tissue and cell debris, a phase that is followed by anti-inflammatory Ly-6C^low^ macrophages, which promote the resolution of inflammation and wound healing [21, 30, 40].

Here, we hypothesize that the upregulation of NEU1 in hearts exposed to I/R may promote inflammation, arrhythmias, adverse cardiac remodelling, and heart failure. Indeed, we observed a transient upregulation of total neuraminidase activity in the ischemic LV after I/R, which was associated with an increased NEU1, β-GAL, and PPCA and reduced NEU3 expression. Moreover, in mice homozygous for the Neu1a allele (a hypomorphic allele (hNEU1) with moderate reduction of NEU1 expression in some tissues, caused by promoter mutations generating a site for the transcriptional repressors Nkx3-1 and 3–2 and additional mutations in the coding region causing the reduction in neuraminidase activity [5]), a shift from pro-inflammatory monocytes to anti-inflammatory macrophages and better cardiac function were observed after I/R injury compared with WT mice homozygous for the wildtype NEU1b allele. Further analyses using BM transplantation experiments revealed that NEU1 in invading immune cells and locally in the heart contributes to cardiac dysfunction after I/R. In this regard, after I/R, NEU1 in macrophages promoted enhanced and prolonged inflammation, and in cardiomyocytes NEU1 impaired the expression of the gap junction protein Connexin-43 (CX43) and promoted arrhythmias and LV dysfunction. Taken together, these data point to an adverse effect of an activated NEU1/β-GAL/PPCA complex on the heart after I/R and suggest that targeting this complex may lead to a novel therapeutic strategy to reduce adverse remodelling and heart failure after MI.

## Methods

### Animal experiments

SM/J (JAX stock #000687), 129S2/SvPasCrl, and FVB/NCrl mice were bought from Charles River, Germany. The SM/J mice were backcrossed onto the 129S2/SvPasCrl background for at least ten generations. The genotyping for the SM.129S2 (hNEU1) strain is shown in sFig. 1a. Chimeric FVB/N-Tg(pRP.ExSi-TRE-NEU1) mice were generated by Cyagen Biosciences and crossed with FVB/N-Tg(αMHC-tTA) mice, which carry a tetOff system under the control of the αMHC promoter. NEU1 overexpression was checked in the resulting double transgenic mice by RT-PCR, Western blot, and enzymatic activity assay. The F1 descendant with the strongest overexpression was further bred using a FVB/NCrl breeding partner to generate the heterozygous FVB/N-Tg(pRP.ExSi-TRE-NEU1)6-Tg(αMHC-tTA) mouse strain (hereafter NEU1-Tg, N1-Tg for double transgenic progeny). The generation of the mouse strain FVB/N-Tg(pRP.ExSi-TRE-NEU3)12-Tg(αMHC-tTA) was accomplished in an analogous manner (hereafter NEU3-Tg, N3-Tg). For primer sequences see supplementary data. I/R or sham operation was performed as described previously [18] with the exception of the use of 1–2% isoflurane as an anaesthetic. For all operations, adult male mice (9–15 weeks-old) were used.

For transplantations, female BM was isolated by flushing the bones of the hind limbs with cold PBS using a syringe with a 30G cannula. After washing once in PBS, 2.1 × 10^7^ cells, on average, were intravenously injected into lethally irradiated (9 Gy) 8–9 weeks-old male recipients. To prevent infections, Cotrim (ratiopharm, 4.8 mg/ml) was administered via drinking water for three weeks. After 5 ± 1 weeks, I/R injury was induced. Organs were harvested after cervical dislocation. All animal experiments were performed in accordance with German animal protection law and with the European Communities Council Directive 86/609/EEC and 2010/63/EU for the protection of animals used for experimental purposes. All experiments were approved by the Local Institutional Animal Care and Research Advisory Committee and permitted by the local authority LAVES (Niedersächsisches Landesamt für Verbraucherschutz und Lebensmittelsicherheit; Oldenburg, Lower Saxony, Germany).

### Echocardiography

Echocardiographic analysis (Vevo 770, Visual Sonics) was performed as previously described [19] before and 13–14 days after I/R or sham operation. Fractional area change (FAC) was calculated as follows: %FAC = (LVEDA-LVESA)/LVEDA × 100 (LVEDA, left ventricular end-diastolic area; LVESA, left ventricular end-systolic area). Surface ECGs were recorded during the echocardiographic analyses.

### Cardiac immune cell isolation, MACS, flow cytometry

Isolation of infiltrated immune cells was performed as previously described [22] with some modifications. After digestion of pooled WT hearts or spleens with collagenase II (245 U/ml, Worthington) for 30 min at 37 ℃, the filtered cell suspension was incubated with anti-CD11b MicroBeads (Miltenyi Biotec) and purified by magnetic sorting (MACS) as recommended by the manufacturer. Afterwards, the enrichment was checked by flow cytometry using anti-CD11b-FITC (1:10, Beckman Coulter) and anti-CD45-APC (1:20, BD Biosciences) antibodies in 100 µl PBS. Isolated CD11b^+^ cells were lysed using TRIzol^®^ Reagent for gene expression analysis. For characterization of the cell infiltrate after I/R, the right ventricles were removed and the weighted LV were individually digested in 1 ml collagenase II solution. The single-cell suspensions were blocked with purified anti-mouse CD16/CD32 (1 µg/10^6^ cells) for 5 min at 4 ℃ and split before incubation either with anti-CD45-PE (1:100, BioLegend) or with a mix of anti-lineage (Lin) antibodies (CD90.2-PE, B220-PE, CD49b-PE, NK1.1-PE, Ly-6G-PE; all 1:200, BD Biosciences), anti-CD11b-APC (1:20, BioLegend), anti-Ly-6C-PE/Cy7 (1:20, BD Biosciences) and anti-F4/80-FITC (1:20, BioLegend) in 100 µl PBS for 20 min at 4 ℃ in the dark. Appropriate conjugated IgG proteins were used as negative controls. Pro-inflammatory macrophages were defined as Lin^−^CD11b^+^-F4/80^+^Ly-6C^high^ and anti-inflammatory macrophages as Lin^−^CD11b^+^-F4/80^+^Ly-6C^low^ according to Korf-Klingebiel et al*.* [25]. All antibody clones are provided in the supplementary data file. The total amount of CD45^+^ cells per LV was extrapolated and adjusted for tissue weight. FACS analyses were performed using a FACSCalibur maschine (BD), the gating strategy is provided in sFig.2a-c and the comparison of F4/80-Ly-6C and isotype control is shown in sFig.2d.

### RNA isolation and qRT-PCR

Total RNA was isolated using TRIzol^®^ Reagent (Life Technologies) in accordance with the manufacturer’s instructions. For cDNA synthesis with Superscript III (Invitrogen), 2 µg of total RNA and random hexamer primers (Sigma-Aldrich) were used as recommended by the manufacturer. Semi-quantitative real time-PCR was carried out in triplicates utilizing SYBR Green qPCR 2 × Master Mix (Thermo Scientific) in the AriaMX System (Agilent Technologies). The evaluation was done according to the 2-ΔΔCt method [34] with normalization to 18S rRNA, BCL-2, HPRT or TPT1 expression. Primer sequences can be found in the supplementary data.

### RNASeq and bioinformatics

Details on RNASeq and bioinformatics are provided in the supplementary methods.

### Analysis of neuraminidase activity

2′-(4-methylumbelliferyl)-α-D-N-acetylneuraminic acid sodium salt hydrate (4-MU-NANA) (Sigma-Aldrich) was used as an artificial substrate to determine neuraminidase activity. LV tissue was quickly homogenized in the water on ice (100 mg/ml), before 50 µl of the homogenate (in duplicates) were incubated in 50 mM sodium acetate with 0.1% Triton X-100, pH 4.4 and supplemented with 5% protease inhibitor cocktail (Roche Diagnostics) (final volume 200 µl) for 10 min at room temperature. To measure neuraminidase activity in BM cells, the latter were isolated by flushing the bones with PBS. At least 5 × 10^6^ cells were resuspended in 50 µl PBS and incubated as described above. After the addition of 25 µl 4-MU-NANA (final concentration 0.25 mM) samples were incubated for 1 h at 37 ℃ with mixing every 15 min. The reaction was stopped by adding 1 ml 133 mM glycine (Carl Roth), 60 mM sodium chloride (Baker), and 42 mM sodium carbonate (Sigma-Aldrich), pH 10.3. The fluorescence of released 4-MU was measured in 200 µl of supernatant with a fluorometer (excitation at 355 nm, emission at 460 nm) after centrifugation for 3 min at full speed. As standard, a serial dilution of 4-MU (Sigma-Aldrich) in stop buffer (3–0.05 nmole) was used, and one unit (U) of neuraminidase activity was defined as 1 nmole 4-MU released over 1 h at 37 ℃ per mg protein. Protein concentration in the homogenates was determined using a Protein Assay reagent (Bio-Rad) in accordance with the manufacturer’s instructions.

### Protein isolation, SDS-PAGE, Western blot

The total protein was isolated by lysing frozen LV tissue in RIPA buffer supplemented with 10 µM 1,4-dithiothreitol (Sigma-Aldrich) and protease and a phosphatase inhibitor cocktail (Roche Diagnostics) on ice. For SDS-PAGE 50 µg protein was loaded and transferred to a nitrocellulose membrane after separation. The following primary antibodies were used: anti β-GAL (Cell Signaling #27198), anti-CD44 (Abcam ab157107), anti-CX43 (Cell Signaling 3512), anti-iNOS (Cell Signaling #13120), anti-MHC (Abcam ab50967), anti-p-p65^S536^ (Cell Signaling #3033), anti-p65 (Cell Signaling #8284), anti-PPCA (Abcam ab184553), anti-NEU1 (Santa Cruz Biotechnology sc-32936), anti-NEU3 (Santa Cruz sc-134931). Chemiluminescence detection was carried out after incubation with horseradish peroxidase-conjugated anti-rabbit secondary antibody (GE Healthcare) and enhanced chemiluminescence reagents (PerkinElmer) using the ChemiDoc™ MP system (Bio-Rad). Image LabV5.0 software (Bio-Rad) was used for quantification and Ponceau S staining served as a loading control.

### Histology and immunohistochemistry

6 µm cryosections were prepared for LV staining. H&E staining was carried out as previously described [19]. Infiltration by immune cells 14 days after I/R was determined by immunohistochemical staining for CD45 (#550539, BD Biosciences) combined with a counterstain with eosin as previously described [22]. The following antibodies were used for immunostaining: anti-α-actinin (A7811, Sigma-Aldrich), anti-CD68 (ab53444), anti-CX43 (Cell Signaling 3512), anti-F4/80 (Cell Signaling 30325), anti-GSL I IsolectinB4 (Vector B-1205), anti-NEU1 (sc-32936, Santa Cruz Biotechnology), Fluorescein-labelled anti-wheat germ agglutinin (WGA) (Vector Laboratories Inc. FL-1021), Fluorescein-labelled Avidin D (Vector A-2001) and anti-mouse IgG-Alexa Fluor^®^ 488 and anti-rabbit IgG-Cy3 (both from Jackson ImmunoResearch). For nuclear staining, Hoechst (Thermo Fisher Scientific Inc.) was used.

### Immunoprecipitation

LV tissue (250 μg protein) of WT and N1-Tg mice was used for immunoprecipitation. Samples were incubated for three hours at 4 ℃ with anti-PPCA antibody (ab184553, Abcam) under rotation. Fifty microliters of protein A/G PLUS agarose (sc-2003, Santa Cruz Biotechnology) were added to each mixture and incubated overnight at 4 ℃ under rotation. After centrifugation for 30 s at 3000 rpm, supernatants were discarded and the beads were washed four times with PBS. After centrifugation, the beads were resuspended in 40 ml loading buffer and boiled at 99 ℃ for 5 min. The beads were centrifuged for 2 min at 4 ℃ and the supernatants were subjected by SDS-PAGE for Western blot analysis.

### Microscopy

Images were taken either with a microscope Axio Observer 7 or with a Leica SP8 inverted confocal microscope at the Core Facility for Laser Microscopy at Hannover Medical School.

### Statistical analysis

All data are presented as mean ± SD. GraphPad Prism 5.0 for Mac OS X software was used for statistical analyses. For normally distributed values (Kolmogorov–Smirnov test) unpaired, two-tailed *t* test (partially with Welch’s correction) or One- or Two-Way-ANOVA with Bonferroni post-tests were used to analyze group differences. In all other cases, a non-parametric test was applied as indicated in the captions. A *P* value of < 0.05 was considered statistically significant.

## Results

### Neuraminidase activity and expression of neuraminidase isoforms in the adult mouse heart and spleen after exposure to I/R

**Fig. 1:**
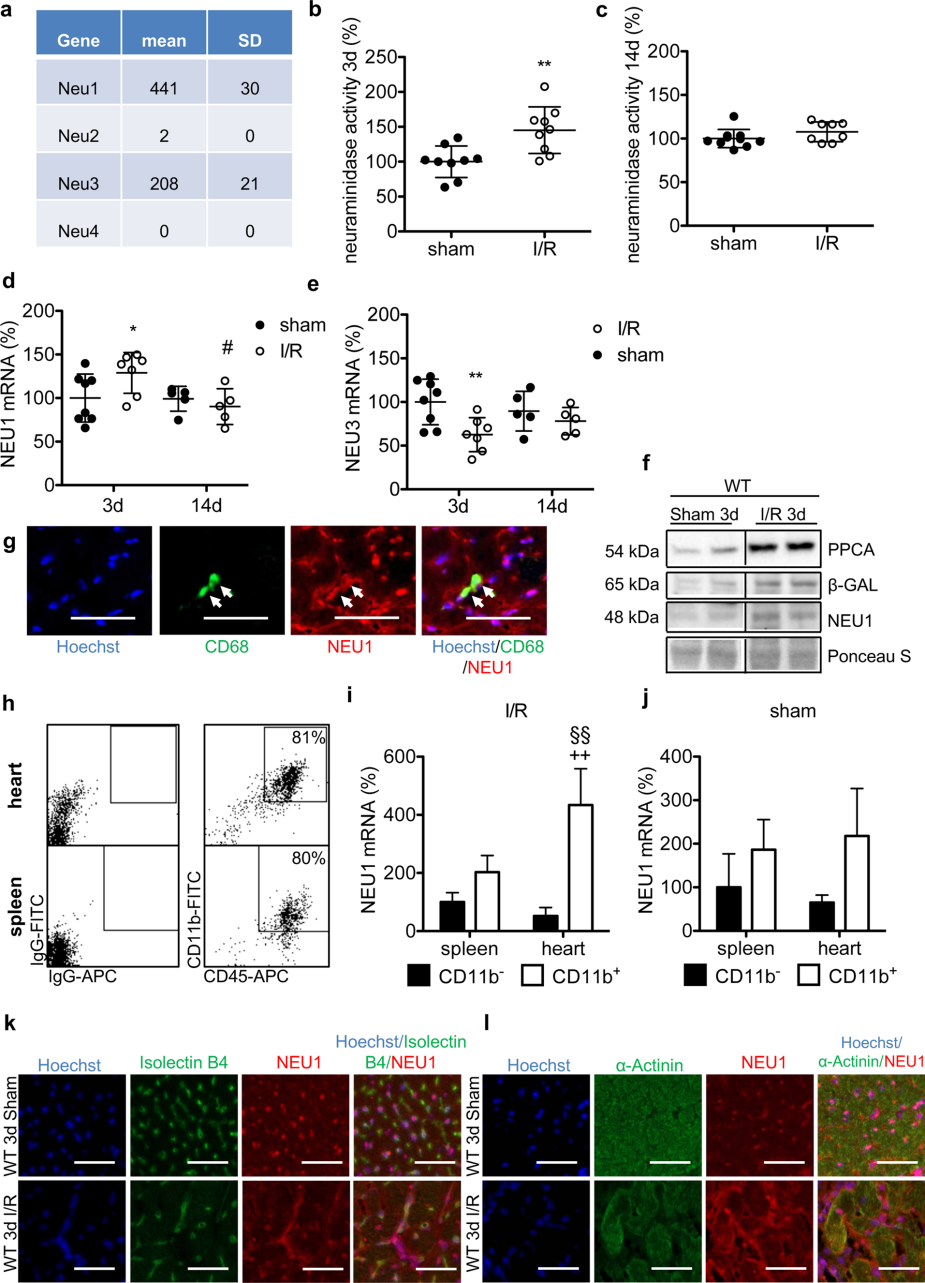
Induced NEU1 expression and activity in the LV after I/R. **a** Expression of NEU1-4 was analysed by RNAseq in RNA isolated from LV tissue from 3 pools (*N* = 3–4 per pool) of WT male mice, aged 3 months. Relative neuraminidase activity in ischemic LV of 129S2/SvPasCrl mice **b** 3 days and **c** 14 days after I/R, respectively, in comparison with sham-operated controls after normalization to protein content. Sham 3 days *N* = 9; I/R 3 days *N* = 9; sham 14 days *N* = 10; I/R 14 days *N* = 8. Relative NEU1 (**d**) and NEU3 (**e**) mRNA expression in ischemic LV of 129S2/SvPasCrl mice 3 days and 14 days after I/R, respectively, as compared with sham-operated controls after normalization to TPT1. Sham 3 days *N* = 8; I/R 3  days *N* = 7; sham 14 days *N* = 5; I/R 14 days *N* = 5. **f** Western blot analysis of NEU1 cofactors PPCA and β-GAL 3 days after I/R compared with sham WT LV. *N* = 2. **g** Representative immunostaining of NEU1 (red), CD68 (green) and nuclear Hoechst (blue) staining in WT LV 3 days after I/R. Scale bar: 50 µm. **h** Representative dot plots of flow cytometric analysis of isolated CD45^hi^CD11b^hi^ cells from 129S2/SvPasCrl hearts 3 days after I/R. Relative NEU1 mRNA expression in CD11b^+^ and CD11b^−^ cells, respectively, isolated from spleens or hearts of 129S2/SvPasCrl mice 3 days after I/R (**i**) or sham (**j**) operation, normalized to 18S. Data in **h–j** derive from 3 (sham) and 4 (I/R) independent experiments with 4–9 pooled hearts and spleens for each experiment. Representative immunostaining of NEU1 (red) upregulation in the heart 3 days after I/R in different cell types including epithelial cells (Isolectin B4 green) (**k**) and cardiomyocytes (α-actinin green) (**l**). Scale bar: 50 µm. All values are depicted as mean ± SD. Statistical analysis was done using Two-Way-ANOVA with Bonferroni post-test (**d**, **e**, **i**, **j**) and unpaired, two-tailed *t* test (**b**, **c**), respectively. *P < 0.05 and **P < 0.01 vs. sham; ^#^*P* < 0.05 vs. 3 days; ^§§^*P* < 0.01 vs. spleen; ^+ +^*P* < 0.01 vs. CD11b^−^

The expression levels of the four different NEU isoforms (NEU1-4) in LV tissue from healthy adult wildtype (WT) male mouse hearts (C57BL/6J) were analysed by RNAseq, which revealed that only NEU1 and NEU3 were substantially expressed in the heart (Fig. 1a).

Time course analyses in the ischemic area of the LV revealed that neuraminidase activity was increased after ischemia for 1 h and reperfusion for 3 days, and returned to the same levels as those of corresponding sham-operated mice after 14 days of I/R (Fig. 1b, c). In parallel, a transient upregulation of NEU1 and a down-regulation of NEU3 mRNA levels with a peak at 3 days after I/R was observed (Fig. 1d, e). Moreover, the NEU1 cofactors PPCA and β-GAL were also upregulated 3 days after I/R in WT LV (Fig. 1f). NEU1 was co-immunoprecipitated with β-GAL and PPCA 3 days after I/R (sFig. 3a).

High NEU1 immunohistochemical staining was observed in monocytes/macrophages in the ischemic LV 3 days after I/R (Fig. 1g). CD45^hi^CD11b^hi^ (myeloid) and CD45^hi^CD11^low^ (lymphoid) cells were isolated from the heart and the spleen of mice, which had either been exposed to 3 days I/R or undergone sham operation (Fig. 1h). NEU1 mRNA levels were significantly higher in the CD45^hi^/CD11b^hi^ myeloid population (monocytes/macrophages) isolated from the LV compared with those isolated from the spleen of mice with I/R operation, whereas no difference was observed in sham-operated mice (Fig. 1i, j). In turn, the ratio of NEU1 expression in CD45^hi^CD11b^low^ (lymphoid) cells of LV and spleen were similar 3 days after I/R and comparable with that in sham-operated mice (Fig. 1i, j).

Immunohistochemical analysis revealed that 3 days after I/R, there was an increased signal of NEU1 staining in cardiomyocytes with a clear accumulation at the cell membrane compared with sham-operated mice, while no difference in NEU1 staining was observed in endothelial cells (Fig. 1k, l).

### Neu1-hypomorphic mice exhibit lower neuraminidase activity and lower inflammatory signalling after I/R

**Fig. 2:**
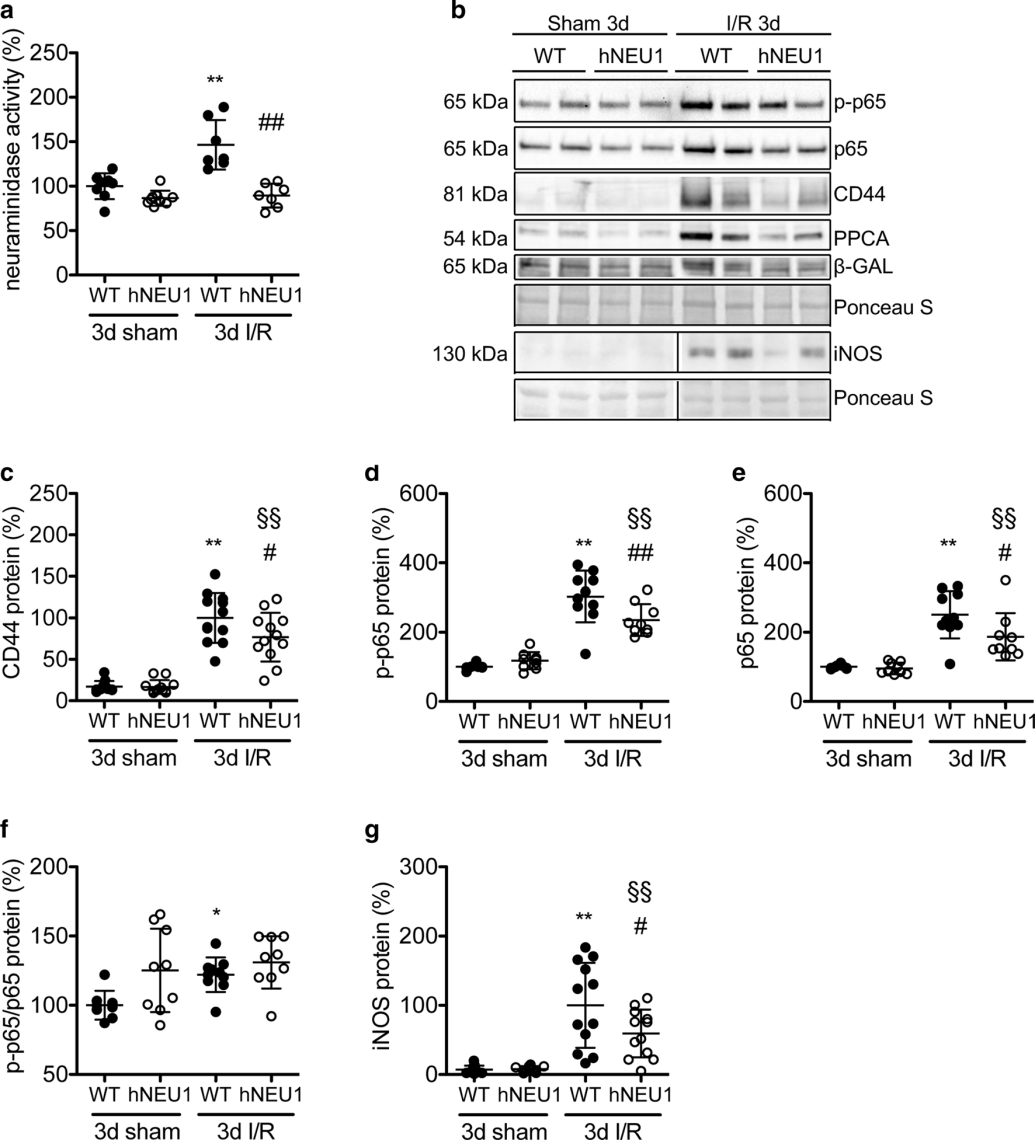
Blunted iNOS, p65 and CD44 induction in NEU1-hypomorphic (hNEU1) mice after I/R. **a** Relative neuraminidase activity in LV of hNEU1 and WT mice 3 days after I/R or sham operation after normalization to protein concentration. WT sham *N* = 8; hNEU1 sham *N* = 9; WT I/R *N* = 7; hNEU1 I/R *N* = 7. **b** Representative Western blots and relative quantification (**c-g**), respectively, of the total (WT sham *N* = 8; hNEU1 sham *N* = 9; WT I/R *N* = 10; hNEU1 I/R *N* = 9) and phosphorylated (S536) p65 (WT sham *N* = 8; hNEU1 sham *N* = 9; WT I/R *N* = 10; hNEU1 I/R N = 9) and the ratio of phosphorylated to total p65 (WT sham *N* = 8; hNEU1 sham *N* = 9; WT I/R *N* = 10; hNEU1 I/R *N* = 9), CD44 (WT sham *N* = 11; hNEU1 sham *N* = 12; WT I/R *N* = 12; hNEU1 I/R *N* = 12) and iNOS (WT sham *N* = 11; hNEU1 sham *N* = 12; WT I/R *N* = 12; hNEU1 I/R *N* = 12) protein levels in the ischemic LV of hNEU1 and WT mice 3 days after sham or I/R injury normalized to Ponceau loading control. All values are depicted as mean ± SD. Statistical analysis was done using or Two-Way-ANOVA with Bonferroni post-test (**a**, **c–g**). *P < 0.05 and **P < 0.01 WT I/R 3 days vs. WT sham 3 days; ^§§^*P* < 0.01 hNEU1 I/R 3 days vs. hNeu1 sham 3 days; ^#^*P* < 0.05 hNEU1 I/R 3 days vs. WT I/R 3 days

Male mice homozygous for a hypomorphic *neu1a* allele (hNEU1, 129S2/SvPasCrl background) displayed normal cardiac function at 3 months of age (sTable 1). NEU1 protein was comparable in the hearts of sham-operated hNEU1 and WT mice (sFig. 1b). Despite similar protein levels, the neuraminidase activity in LV tissue was reduced in hNEU1 compared with WT mice (sFig. 1c). When exposed to I/R for 3 days, WT mice displayed a robust increase in neuraminidase activity, while in hNEU1 mice the increase was absent (Fig. 2a). HNEU1 mice showed also less induction of NEU1 cofactors PPCA and β-GAL in the ischemic LV tissue, which was associated with reduced protein expression of CD44, iNOS, and the NF-κB subunit p65 and lower phosphorylation of p65 compared with WT mice (Fig. 2b-g). When comparing the ratio of phosphorylated p65 to total p65 protein, there was a significant increase in WT mice 3 days after I/R compared to sham-operated animals, while there was no increase in hNEU1 mice after I/R (Fig. 2f).

### Neu1-hypomorphic mice display a shift from pro-inflammatory to anti-inflammatory macrophages 3 days after I/R, better cardiac function and less impaired CX43 expression 14 days after I/R

**Fig. 3:**
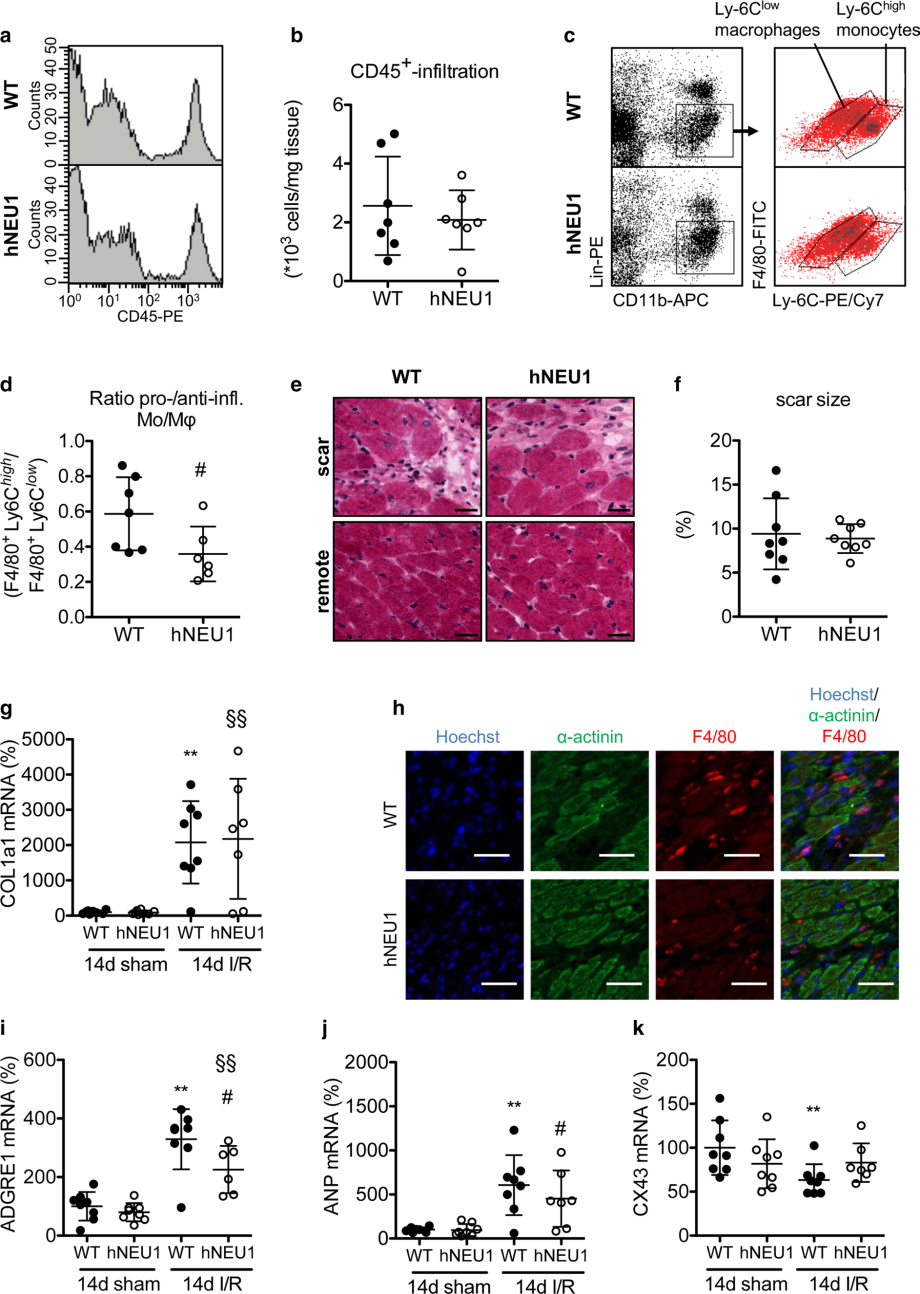
Analysis of infiltrated immune cells and infarct sizes in NEU1-hypomorphic mice after I/R. **a, b** Representative flow cytometry histograms and quantification of infiltrated CD45^+^ cells in LV of hNEU1 and WT mice 3 days after I/R. *N* = 7. **c** Representative dot plots of flow cytometric analysis of infiltrated pro-inflammatory Lin^−^CD11b^+^F4/80^+^Ly-6C^high^ and anti-inflammatory Lin^−^CD11b^+^F4/80^+^Ly-6C^low^ macrophages in LV of hNEU1 and WT mice 3 days after I/R. **d** Ratio of F4/80^+^Ly-6C^high^ and F4/80^+^Ly-6C^low^ macrophages per mouse 3 days after I/R. WT *N* = 7; hNEU1 *N* = 6. **e** Representative images of H&E stained ischemic (scar) and remote LV of hNEU1 and corresponding WT mice 14 days after I/R. Scale bar: 20 µm. **f** Quantification of scar patches was done by measuring scar area in comparison with the whole LV area in % on 43 and 40 serial slides, respectively. *N* = 8. **g** COL1a1 mRNA expression in the ischemic LV of WT and hNEU1 mice 14 days after I/R, normalized to TPT1. WT sham *N* = 8, hNEU1 sham *N* = 8; WT I/R *N* = 8; hNEU1 I/R *N* = 7. **h** Representative immunohistochemistry staining of F4/80 (ADGRE1) (red) and α-actinin (green) in ischemic LV of hNEU1 and WT mice 14 days after I/R. Scale bar: 50 µm. **i** ADGRE1 (WT sham *N* = 8; hNEU1 sham *N* = 8; WT I/R *N* = 8; hNEU1 I/R *N* = 6) and (j) ANP (WT sham *N* = 8, hNEU1 sham *N* = 8; WT I/R *N* = 7; hNEU1 I/R *N* = 7) and (k) CX43 (WT sham *N* = 8, hNEU1 sham *N* = 8; WT I/R *N* = 8; hNEU1 I/R *N* = 7), respectively, mRNA expression in the ischemic LV of WT and hNEU1 mice 14 days after I/R and sham operation, normalized to TPT1. All values are depicted as mean ± SD. Statistical analysis was done using unpaired two-tailed *t* test with Welch’s correction (**b**, **d**, **f**) and Two-Way-ANOVA with Bonferroni post-test (**g**, **i–k**). For panel d: ^#^*P* < 0.05 hNEU1 I/R 33 daysvs. WT I/R 3 days; for panel f, g, i-k: **P < 0.01 WT I/R 14 days vs. WT sham 14 days; ^§§^*P* < 0.01 hNEU1 I/R 14 days vs. hNeu1 sham 14 days ^#^*P* < 0.05 hNEU1 I/R 14 days vs. WT I/R 14 days

FACS analysis showed no difference in the total number of invading leukocytes (CD45^+^ cells) in the hearts of hNEU1 and WT mice 3 days after I/R (Fig. 3a, b). However, the FACS analysis also revealed that the ratio of pro-inflammatory Lin^−^CD11b^+^-F4/80^+^Ly-6C^high^ to anti-inflammatory Lin^−^CD11b^+^-F4/80^+^Ly^−^6C^low^ macrophages was lower in hNEU1 compared with WT mice 3 days after I/R (Fig. 3c, d).

Fourteen days after I/R, hNEU1 mice displayed similar infarct scar sizes, a similar increase in the expression of Collagen 1A1 (COL1a1), and no difference in heart weight (HW), body weight (BW), and the HW/BW ratio compared with WT mice (Fig. 3e-g, Table 1). However, hNEU1 mice displayed significantly better LV function compared with WT mice (Table 1). The increase in the expression of the macrophage marker ADGRE1 (F4/80) was lower in hNEU1 mice compared with WT LV 14 days after I/R, which was also confirmed by immunohistochemical analysis (Fig. 3h, i). Both genotypes displayed increased expression of the cardiac stress marker Atrial Natriuretic Peptide (ANP) (Fig. 3j), while a slight but significant decrease in the expression of the Gap junction CX43 was observed in the ischemic LV of WT, but not of hNEU1 mice 14 days after I/R (Fig. 3k).

**Table 1:** Cardiac function and morphometry in hNEU1 mice 14 days after sham or I/R operation

	Sham		I/R	
	WT *N* = 14	hNEU1 *N* = 12	WT *N* = 12	hNEU1 *N* = 13
FAC (%)	62 ± 5	60 ± 4	42 ± 14**	50 ± 8^§§#^
LVEDA (cm^2^)	15.6 ± 1.5	16.7 ± 1.4	19.8 ± 2.4**	19.1 ± 1.9^§§^
LVESA (cm^2^)	6.0 ± 1.1	6.7 ± 1.2	11.8 ± 4.0**	9.7 ± 2.1^§§^
HR (bpm)	543 ± 36	536 ± 30	528 ± 27	524 ± 34
BW (g)	27 ± 3	25 ± 3	26 ± 3	28 ± 2
HW (mg)	111 ± 11	110 ± 14	115 ± 12	114 ± 11
HW/BW (mg/g)	4.2 ± 0.3	4.4 ± 0.5	4.4 ± 0.3	4.1 ± 0.3

All values are depicted as mean ± SD. Statistical analysis was done using Two-Way-ANOVA with Bonferroni post-test

*FAC* Fractional area change, *LVEDA* left ventricular end-diastolic area, *LVESA* left ventricular end-systolic area, *HR* heart rate, bpm beats per minute, *BW* body weight, *HW* heart weight

**P < 0.01 WT I/R vs. WT sham

^§§^*P* < 0.01 hNEU1 I/R vs. hNEU1 sham

^#^*P* < 0.05 hNEU1 I/R vs. WT I/R

### Bone marrow cell transplantation reveals that NEU1 in invading immune cells and locally in the myocardium impacts on cardiac function after I/R

**Fig. 4:**
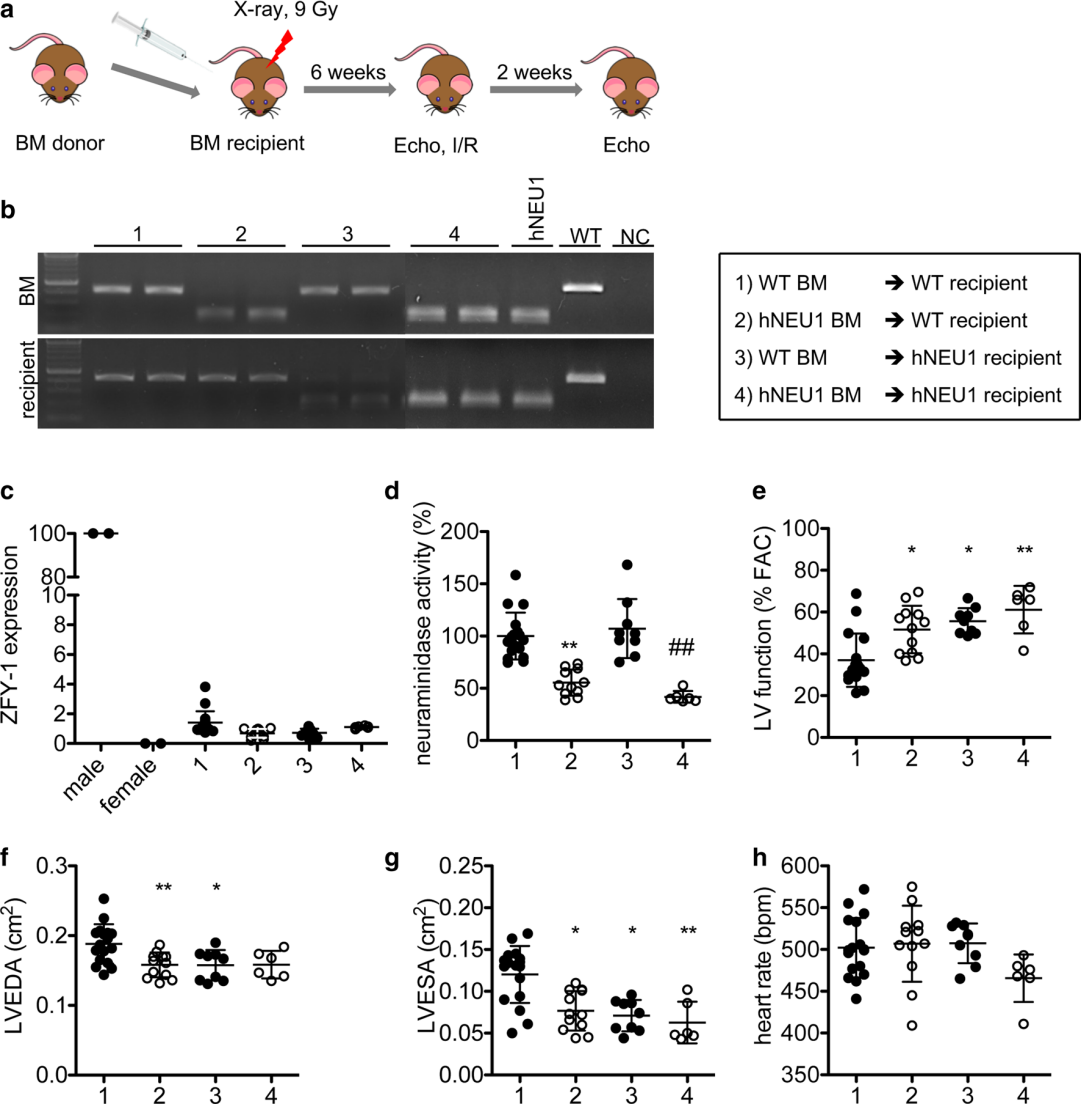
Bone marrow transplantation experiments reveal the impact of NEU1 in invading haematopoietic cells and in the myocardial tissue on heart function. (**a**) Experimental setup of BM transplantation experiments between male WT and hNEU1 mice. (**b**) Representative genotyping of transplanted donor BM and appropriate recipient 14 days after I/R for the 240C→T point mutation [5]. (**c**) Relative expression of the *Zfy-1* gene located on the Y chromosome in explanted female BM cells 14 days after I/R, normalized to *Bcl-2*. (male *N* = 2; female *N* = 2; 1 *N* = 17; 2 *N* = 12; 3 *N* = 9; 4 *N* = 6). (**d**) Relative neuraminidase activity (mean of WT BM transplanted in WT mice was set as 100%) in explanted BM cells 14 days after I/R after normalization to protein concentration. (1 *N* = 17; 2 *N* = 11; 3 *N* = 9; 4 *N* = 6). Arrow indicates the direction of the transplantation of the respective BM cells into the recipient. LV function (**e**), LVEDA (**f**), LVESA (**g**) and heart rate (**h**) of BM transplanted mice 14 days after I/R injury. 1 *N* = 17; 2 *N* = 12; 3 *N* = 9; 4 *N* = 6. All values are depicted as mean ± SD. Statistical analysis was done using unpaired two-tailed *t* test for WT and hNEU1 recipients separately (**d**) and One-Way-ANOVA with Bonferroni post-test (LVEDA) and Kruskal–Wallis test with Dunn’s post-test (FAC, LVESA, HR), respectively. *P < 0.01 and **P < 0.05 vs. WT-BM → WT recipient (1); ^#^*P* < 0.05 vs. WT-BM → hNEU1 recipient. *NC* negative control

To analyse whether the beneficial effects of lower NEU1 expression and activity in hNEU1 mice derive from invading immune cells or locally from the heart, BM transplantation experiments were performed between WT and hNEU1 mice (Fig. 4a). Successful substitution of male recipient’s BM by female donor cells was confirmed in explanted BM cells at the end of the experiment by the absence of male gene *Zfy-1* expression and neuraminidase activity in the BM (Fig. 4b-d). There was no difference in survival rates between the groups after transplantation and during the recovery time (6 weeks). Thereafter, transplanted mice were exposed to I/R. Fourteen days after I/R, WT mice with hNEU1-BM, but also hNEU1 mice with WT-BM, showed significantly better LV function compared with WT mice reconstituted with WT-BM. LV function tended to be highest in hNEU1 mice transplanted with hNEU1-BM (Fig. 4e-g). No significant differences were observed in the heart rate (Fig. 4h) across all conditions. These data suggest that NEU1 in invading immune cells, but also in cardiomyocytes, promotes heart failure after I/R.

### Cardiomyocyte-specific overexpression of NEU1 has no effect on cardiac function and inflammation at baseline and after I/R

**Fig. 5:**
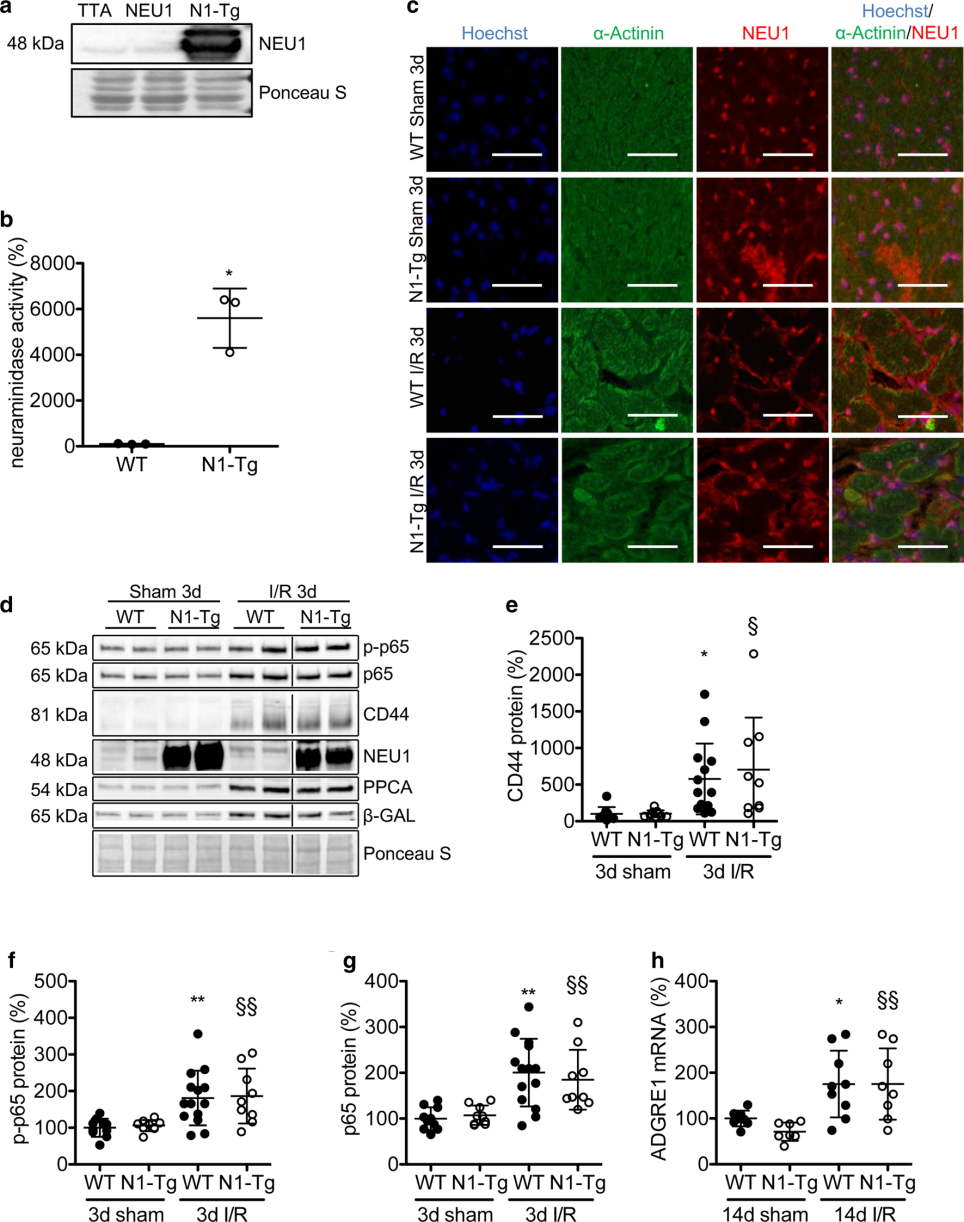
Cardiomyocyte-specific overexpression of NEU1 does not promote inflammation after I/R. **a** Representative Western blot shows NEU1 overexpression in the LV of a N1-Tg mouse as compared with WT hearts and Ponceau staining for loading control. **b** Neuraminidase activity in the LV of N1-Tg and corresponding WT mice. *N* = 3. **c** Microscopy of representative immunostaining of cardiomyocyte-specific NEU1 overexpression (red), α-actinin (green) and nuclear Hoechst staining (blue) in LV of N1-Tg and corresponding WT mice 3 days after sham or I/R operation. Scale bar: 50 µm. **d** Representative Western blot in ischemic LV of N1-Tg and WT mice 3 days after sham or I/R injury. Relative quantification (**e–g**) of total and phosphorylated (S536) p65 and CD44, respectively, in ischemic LV of N1-Tg and WT mice 3 days after sham or I/R injury normalized to Ponceau loading control. WT sham *N* = 10; N1-Tg sham *N* = 9; WT I/R *N* = 14; N1-Tg I/R *N* = 9. **h** ADGRE1 mRNA expression in the ischemic LV of N1-Tg and WT mice 14 days after I/R and sham operation, normalized to 18S. WT sham *N* = 8; N1-Tg sham *N* = 7; WT I/R *N* = 9; N1-Tg I/R *N* = 8. All values are depicted as mean ± SD. Statistical analysis was done using unpaired, two-tailed *t* test (**b**) and Two-Way-ANOVA with Bonferroni post-test (**d**, **f–h**). For panel b: *P < 0.5 N1-Tg vs. WT mice, for panel **e–g**: *P < 0.05 and **P < 0.01 WT I/R 3 days vs. WT sham 3 days, for panel **h**: *P < 0.05 WT I/R 14 days vs. WT sham 14 days; for panel **e–g**: ^§^*P* < 0.05 and ^§§^*P* < 0.01 N1-Tg I/R 3 days vs. N1-Tg sham 3 days, for panel **h**: ^§§^*P* < 0.01 N1-Tg I/R 14 days vs. N1-Tg sham 14 days

To analyse the effect of increased NEU1 expression in cardiomyocytes after I/R, we generated a mouse with a cardiomyocyte-specific NEU1 overexpression combining an inducible NEU1 transgene (FVB/N-Tg(pRP.ExSi-TRENEU1)) with a tetOff system under the control of the α-MHC promoter (Tg(αMHC-tTA)). The double transgenic progeny N1-Tg was born at the expected Mendelian ratio and, at the age of 3 months, had a robust overexpression of NEU1 in cardiomyocytes and a significantly increased neuraminidase activity in the LV (Fig. 5a-c). Echocardiography in adult male mice showed no impact of NEU1 overexpression on cardiac function, although the LV end-diastolic area (LVEDA) was larger compared with corresponding WT mice (NEU1, TTA or double negative mice, all termed WT mice, sTable 2).

Exposing N1-Tg mice to I/R for 3 days led to increased localization of NEU1 at the cardiomyocyte membrane, which was associated with an increase in the expression of NEU1 cofactors PPCA and β-GAL (Fig. 5c, d). Inflammatory markers CD44, the NF-κB subunit p65, phosphorylation of p65 and ADGRE1 were upregulated to a similar degree in N1-Tg mice and WT mice 3 days after I/R compared with corresponding shams (Fig. 5d-h).

### Cardiomyocyte-specific overexpression of NEU1 has no effect on scar size, but leads to cardiac hypertrophy, impaired CX43 expression, and localization, and reduced LV function 14 days after I/R

**Fig. 6:**
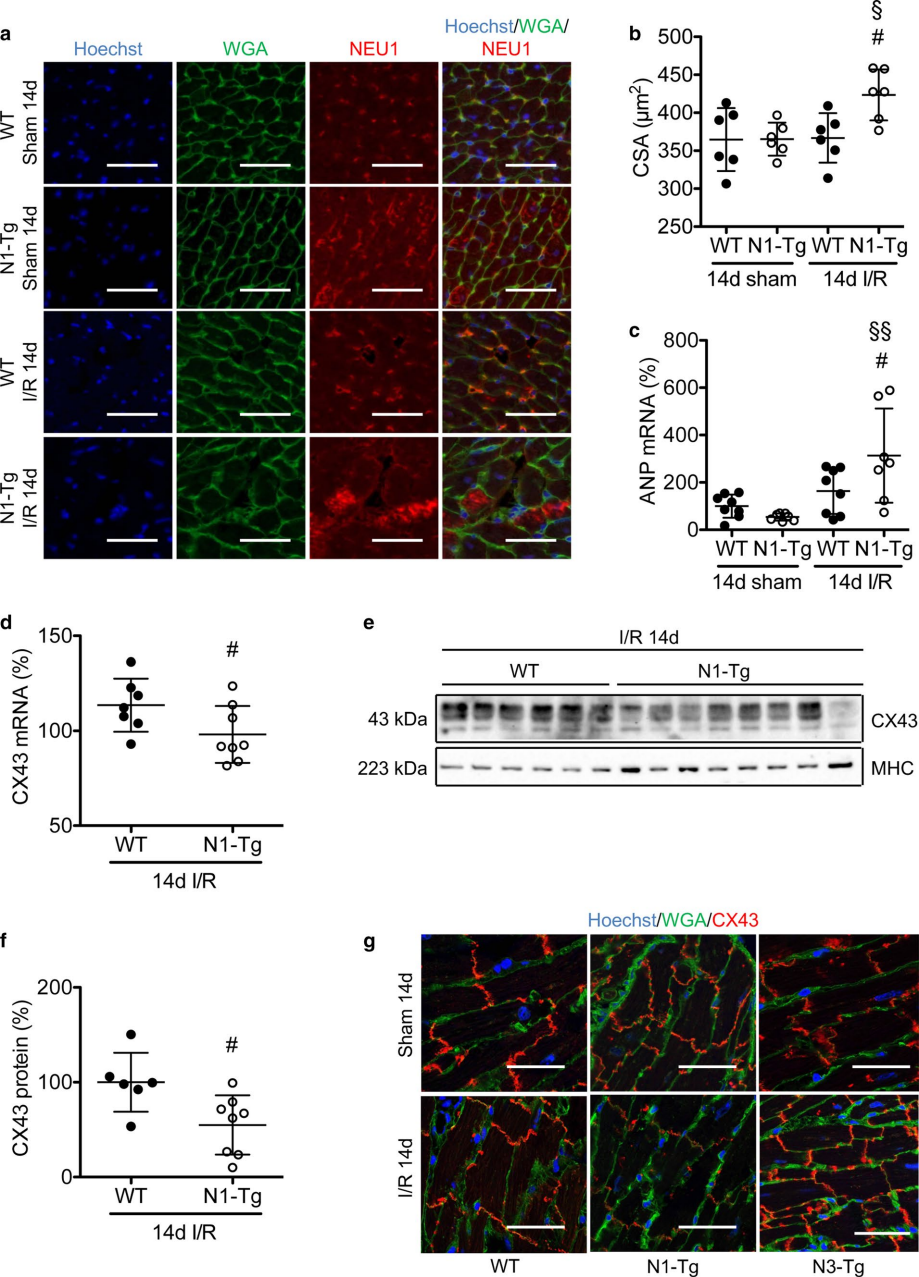
Cardiomyocyte-specific overexpression of NEU1, but not of NEU3 affects localization of CX43 and triggers cardiomyocyte hypertrophy. **a** Representative microscopy of immunohistochemical staining for WGA (green), NEU1 (red) and nuclear Hoechst (blue) on LV of N1-Tg and corresponding WT mice 14 days (14d) after sham and I/R injury. Scale bar: 50 μm. **b** Analysis of the cross-sectional area (CSA) of cardiomyocytes in WT and N1-Tg mice. Quantification was done by measuring the CSA of 29–57 cardiomyocytes per heart (*N* = 6 in each group). **c** ANP mRNA expression in the ischemic LV of WT and N1-Tg hearts 14 days after I/R or sham operation, normalized to 18S. WT sham *N* = 8; N1-Tg sham *N* = 7; WT I/R *N* = 8; N1-Tg I/R *N* = 7. **d** Relative CX43 mRNA expression in WT and N1-Tg 14 days after I/R normalized to HPRT. WT I/R *N* = 7; N1-Tg I/R *N* = 8. **e** Western blot of CX43 protein expression in N1-Tg and corresponding WT LV 14 days after I/R injury, normalized to MHC and **f** quantification of CX43 protein levels in the ischemic LV of N1-Tg and WT mice 14 days after I/R injury normalized to heavy chain cardiac myosin (MHC). WT I/R *N* = 6; N1-Tg I/R *N* = 8. **g** Confocal microscopy of immunohistochemical double staining on cryosections for CX43 (red), WGA (green) and nuclear Hoechst staining (blue) of WT, N1-Tg and N3-Tg hearts 14 days after I/R or sham operation. Scale bars: 50 μm. All values are depicted as mean ± SD. Statistical analysis was done using unpaired, two-tailed *t* test (**d**, **f**) and Two-Way-ANOVA with Bonferroni post-test (**b**, **c**). ^#^*P* < 0.05 N1-Tg I/R 14 days vs. WT I/R 14 days; ^§^*P* < 0.05 and ^§§^*P* < 0.01 N1-Tg I/R 14 days vs. N1-Tg sham 14 days

Immunohistochemical analysis revealed no differences in NEU1 expression 14 days after I/R in N1-Tg and WT mice compared with corresponding sham-operated mice (Fig. 6a). Furthermore, there was no difference in PPCA and β-GAL protein expression in WT mice (sFig. 3b). Morphometric analyses revealed no difference in scar size (sFig. 3c), and similar increases in the expression of COL1a1 and ADGRE1 between N1-Tg and WT mice 14 days after I/R (Fig. 5h and sFig. 3d). However, N1-Tg mice displayed increased HW/BW ratio, increased cardiomyocyte cross-sectional area (CSA), and increased ANP expression, as well as markedly lower expression of CX43 and impairment in its localization in cardiomyocyte’s gap junctions compared with WT mice (Fig. 6a-g). Furthermore, while no significant reduction in LV function in WT mice (FVB/N background) was observed 14 days after I/R compared with sham-operated WT mice, N1-Tg mice displayed a moderate, but significant decrease in LV function compared with corresponding sham-operated mice (Table 2).

**Table 2:** Cardiac function and morphometry in N1-Tg and N3-Tg mice 14 days after I/R

	Sham			I/R		
	WT *N* = 19	N1-Tg *N* = 16	N3-Tg *N* = 8	WT *N* = 8	N1-Tg *N* = 17	N3-Tg *N* = 9
FAC (%)		61 ± 4	58 ± 6	54 ± 3	51 ± 7^§§^	54 ± 5
LVEDA (mm^2^)	18.4 ± 3.0	19.2 ± 2.2	18.0 ± 1.5	18.9 ± 0.9	18.4 ± 2.7	20.0 ± 1.1
LVESA (mm^2^)	7.5 ± 1.3	7.5 ± 1.3	7.6 ± 1.1	8.6 ± 0.7	9.0 ± 0.7^§^	9.3 ± 1.3
HR (bpm)	567 ± 23	565 ± 31	559 ± 18	522 ± 25**	551 ± 31^#^	527 ± 23^++^
BW (g)	28 ± 3	29 ± 3	26 ± 3	28 ± 2	28 ± 3	28 ± 2
HW (mg)	107 ± 11	114 ± 13	108 ± 13	105 ± 9	129 ± 14^§§##^	109 ± 9
HW/BW	3.9 ± 0.4	4.1 ± 0.7	4.2 ± 0.3	3.7 ± 0.2	4.8 ± 0.8^§§##^	3.9 ± 0.2

All values are depicted as mean ± SD. Statistical analysis was done using Two-Way-ANOVA with Bonferroni post-test

*FAC* Fractional area change, *LVEDA* left ventricular end-diastolic area, *LVESA* left ventricular end-systolic area, *HR* heart rate, bpm beats per minute, *BW* body weight, *HW* heart weight

**P < 0.01 WT I/R vs. WT sham

^§^*P* < 0.05 N1-Tg I/R vs. N1-Tg sham

^§§^*P* < 0.01 N1-Tg I/R vs. N1-Tg sham

^++^*P* < 0.01 N3-Tg I/R vs. N3-Tg sham

^#^*P* < 0.05 N1-Tg I/R vs. WT I/R

^##^*P* < 0.01 N1-Tg I/R vs. WT I/R

### Cardiomyocytes-specific overexpression of NEU3 does not reduce CX43 expression nor does it promote heart failure after I/R

To analyse whether cardiac hypertrophy, alterations in CX43, and LV dysfunction in N1-Tg mice after I/R were due to unspecific upregulation of neuraminidase activity in the cardiomyocytes, a mouse with a cardiomyocyte-specific NEU3 overexpression (FVB/N-Tg(pRP.ExSi-TRENEU3; Tg(αMHC-tTA)) was generated. The double transgenic progeny N3-Tg was born at the expected Mendelian ratio and at the age of 3 months, had a robust overexpression of NEU3 in the heart with significantly increased neuraminidase activity (sFig. 4a-c). At the age of 3 months, echocardiography showed normal function in N3-Tg (data not shown). Sham-operated and I/R-operated N3-Tg mice displayed no difference in cardiac dimensions, HW, BW and the HW/BW ratio compared with sham or I/R-operated WT mice (NEU1, TTA or double negative mice, all termed WT mice, Table 2). CX43 expression staining intensity and localization as well as protein expression in N3-Tg LV were comparable with corresponding WT mice 14 days after I/R (Fig. 6g and sFig. 4d).

## Discussion

We report that neuraminidase activity is transiently upregulated in the ischemic part of the left ventricle following I/R with a peak early (3 days) after I/R, where inflammatory reactions are highest, followed by normalization to sham-operated levels in the late phase (14 days) after I/R. We observed that, of the four NEU enzymes in mammalians, only NEU1 and NEU3 are substantially expressed in the adult mouse heart of which NEU1 is up- and NEU3 is down-regulated after I/R. Therefore, we investigated the roles of NEU1 and NEU3 in the heart exposed to I/R and observed that increased NEU1 expression and activation is associated with high inflammation and an increased risk of heart failure, while NEU3 had no such effects.

It is known that marked reduction in NEU1 activity, due to mutations in the gene, leads to sialidosis, an inherited disease characterized by coarse facial features, hepatomegaly, dysostosis multiplex, and developmental delay, myoclonic epilepsy, visual impairment and ataxia [13]. Because of these severe systemic phenotypes associated with substantial loss of NEU1, we used mice with a hypomorphic *Neu1a* allele (hNEU1) to study the role of NEU1 in the heart exposed to I/R. At baseline, NEU1 protein levels were not different in LV tissue from WT and hNEU1 as the transcriptional repressors Nkx3-1 and 3–2 that reduce the expression of the NEU1a allele [5] are not expressed in the heart. However, the neuraminidase activity was reduced at baseline in LV tissue from hNEU1 mice. The overall lower neuraminidase activity in hNEU1 mice led to reduced inflammatory signalling early after I/R and less heart failure in the late phase after I/R. BM transplantation experiments and mice with cardiomyocyte-specific NEU1 overexpression demonstrated that high NEU1 activity in cardiomyocytes as well as in invading inflammatory cells promote cardiac dysfunction after I/R. In line with this observation, the ischemic part of the LV displayed enhanced cardiac NEU1 expression in invading monocytic cells and in cardiomyocytes, while no such differences were observed in endothelial cells.

We, and others, have previously reported that NEU1 plays an important role in different immune cell types [1, 6, 28, 31, 37, 39]. In monocytic and lymphocytic immune cells, NEU1 promotes atherosclerosis and plaque inflammation and thereby acts as a prerequisite for MI [37, 42]. Here, we report that NEU1 in the hematopoietic system plays a role in heart failure after I/R since hNEU1-BM transplanted into WT mice was associated with better LV function after I/R. Further analyses revealed that in the early phase after I/R, NEU1 does not seem to play a role for the invasion of total leukocytes to the heart since the total number of CD45^+^ cells isolated from LV tissue from WT and hNEU1 mice after I/R was similar. However, NEU1 was upregulated in invading monocytic cells, but not in lymphocytes. Moreover, NEU1 was not upregulated in monocytic cells isolated from the splenic reservoir of WT mice early after I/R indicating that the upregulation of NEU1 is specific to monocytic cells invading the injured heart. Since invading monocytes differentiate into macrophages at the site of injury, this observation corresponds with our previous study showing that NEU1 becomes upregulated during the differentiation from monocytes to macrophages [37]. In addition, we also showed that upregulated NEU1 promotes an inflammatory phenotype in monocytic cells with enhanced cytokine production and phagocytosis [37]. In line with the observation of a pro-inflammatory effect of NEU1 in monocytic cells, the present study shows more pro-inflammatory Lin^−^CD11b^+^F4/80^+^Ly-6C^high^ macrophages in relation to anti-inflammatory Lin^−^CD11b^+^F4/80^+^Ly-6C^low^ macrophages in the early phase after I/R in WT mice compared with hNEU1 mice. Moreover, the difference in pro-inflammatory and anti-inflammatory monocytic cells was also reflected by enhanced inflammatory signalling, i.e. NF-κB activation, CD44 and iNOS expression in WT compared with hNEU1 mice early after I/R.

While there were no differences in I/R scar size and COL1a1 expression between WT and hNEU1 mice late after I/R, WT LVs displayed slightly but significantly increased expression of the macrophage marker ADGRE1 compared with hNEU1 LVs, pointing to a persisting higher inflammatory status associated with higher systemic NEU1 expression and activity in WT mice. Thus, in addition to the adverse role of NEU1 in monocytic cells in atherosclerosis [37, 42], NEU1 promotes higher and prolonged inflammation in monocytic cells in the infarcted heart after reperfusion and thereby contributes to heart failure.

Cardiomyocyte-specific overexpression of NEU1 in N1-Tg mice led to diffusely increased NEU1 staining of cardiomyocytes with increased neuraminidase activity, which, however, had no effect on cardiac function and inflammation in basal and sham-operated mice, indicating that upregulation of NEU1 in cardiomyocytes alone is not detrimental for the heart. However, later after I/R, increased cardiomyocyte hypertrophy, lower cardiac function, and decreased and mislocalized CX43 were observed in N1-Tg compared with WT mice. Since no differences in cardiac inflammation in the early and late phases after I/R were observed between N1-Tg and corresponding WT mice, the cardiac phenotype of N1-Tg mice after I/R appears not to be caused by more inflammation, but by specific effects in cardiomyocytes. This idea is also supported by the BM experiments, where the transplantation of WT-BM into hNEU1 mice was associated with improved LV function after I/R.

NEU1 has initially been described as a lysosomal protein with a role in the catabolism of glycosylated proteins [35]. NEU1 needs to form a multienzyme complex with β-GAL and PPCA for catalytical activation and protection from lysosomal degradation [4]. This complex subsequently translocates to the cell surface [4], where the increased neuraminidase activity modulates the regulatory processes of receptor and adhesion molecules [35, 37]. While β-GAL and PPCA were not upregulated in sham-operated mice of either genotype, after I/R β-GAL and PPCA were markedly upregulated in WT and N1-Tg mice, NEU1 was co-precipitated with β-GAL and PPCA, and accumulation of NEU1 at the cell membrane of cardiomyocytes was observed with increased staining in N1-Tg mice, indicating that an active NEU1/β-GAL/PPCA complex in cardiomyocytes has been formed after I/R. This observation may explain why NEU1 overexpression alone in N1-Tg mice has no specific effect, but after I/R, N1-Tg mice develop more cardiomyocyte hypertrophy and display lower cardiac function. Indeed, previous studies reported that an aberrant sialylation or desialylation of the plasma membrane of cardiomyocytes results in disturbed functionality of cardiac ion channels, which may also lead to a higher incidence of cardiac arrhythmias [2, 3, 9–12, 24, 29, 33]. Moreover, enhanced cardiac neuraminidase activity resulted in reduced expression and mislocalization of CX43 in gap junctions of N1-Tg mice compared with WT mice after I/R. Since it is well known that reduced CX43 leads to arrhythmias and dysfunction after I/R injury [7], this feature may at least in part explain enhanced heart failure in N1-Tg mice after I/R.

Interestingly, it has also been shown that lower sialic acid content improves gap junction activities [27]. In this regard, it is known that different NEU enzymes prefer different substrates and have different and sometimes even opposing roles [35]. This notion is supported by our findings in N3-Tg mice, which, despite increased total neuraminidase activity in the heart, did not develop more heart failure after I/R and showed no difference in CX43 expression and localization compared with corresponding WT mice. Of note, in contrast to the C57BL/6 genetic background of hNEU1 mice, I/R did not lead to heart failure in WT mice of the FVB/N genetic background in which NEU1 and NEU3 transgenes were generated and therefore a potential benefit of NEU3 overexpression could not be assessed. NEU1 and NEU3 may also have different roles in different cardiac cell types since NEU3, for example, promotes angiogenesis, while NEU1 inhibits angiogenesis [26]. As these data suggest that unspecific neuraminidase inhibition may have unpredictable effects, specific NEU inhibitors or activators are needed for pharmacological therapies. So far, most available NEU1 inhibitors, such as Zanamivir or N-Acetyl-2,3-dehydro-2-deoxyneuraminsäure (DANA), also target other NEU enzymes, mainly NEU3 [38]. A NEU1-specific inhibitor with bioactivity in human lung endothelial cells and fibroblasts, C9-Butylamid-DANA [23], has recently been developed and might present an interesting candidate to limit I/R injury in the heart.

In conclusion, we demonstrated that, following exposure to I/R in the heart, NEU1 expression and activity are increased in different cell types including cardiomyocytes and invading monocytic cells, and contribute to higher inflammation, hypertrophy, impairment of gap junctions, and heart failure after I/R. These adverse effects are specific to NEU1 since no such effects were observed for NEU3. Therefore, NEU1 specific inhibition and, more specifically, inhibition of the NEU1/β-GAL/PPCA complex could be developed into novel therapies to limit myocardial damage and dysfunction after ischemia and reperfusion injury.

## Electronic supplementary material

Below is the link to the electronic supplementary material.

Supplementary file1 (TIF 22798 kb)

Supplementary file2 (TIF 23374 kb)

Supplementary file3 (TIF 22773 kb)

Supplementary file4 (TIF 22928 kb)

Supplementary file5 (DOCX 32 kb)

## Data Availability

All data and materials used for this study are displayed or can be displayed upon request.
