# Supplementary figures and images for: Neuraminidase-1 promotes heart failure after ischemia/reperfusion injury by affecting cardiomyocytes and invading monocytes/macrophages
Source: Basic Res Cardiol. 2020 Sep 25;115(6):62. doi: 10.1007/s00395-020-00821-z (PMC7519006; doi:10.1007/s00395-020-00821-z)

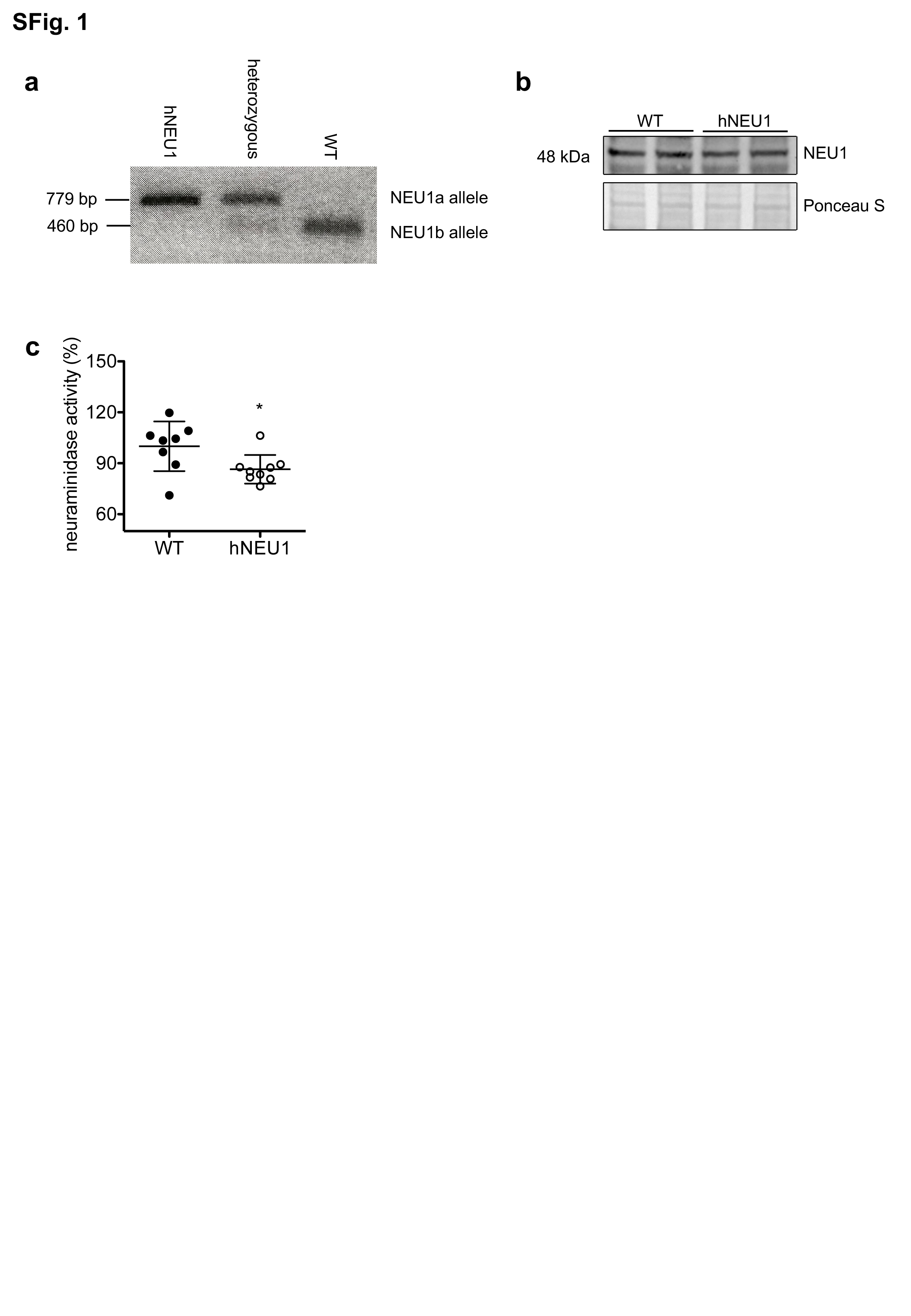

Supplement: Supplementary file 1 — Supplementary file1 (TIF 22798 kb) [file 395_2020_821_MOESM1_ESM.tif]

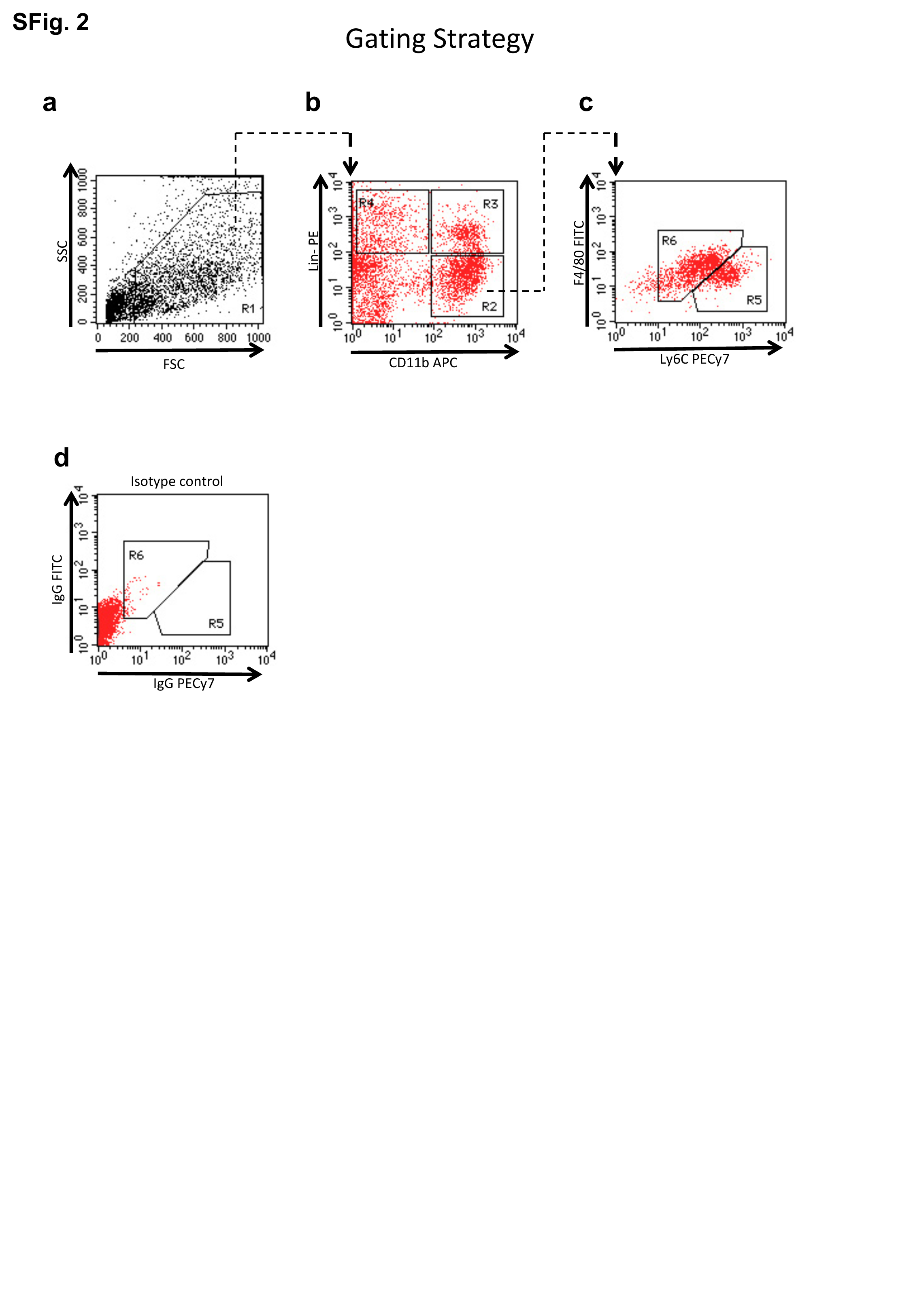

Supplement: Supplementary file 2 — Supplementary file2 (TIF 23374 kb) [file 395_2020_821_MOESM2_ESM.tif]

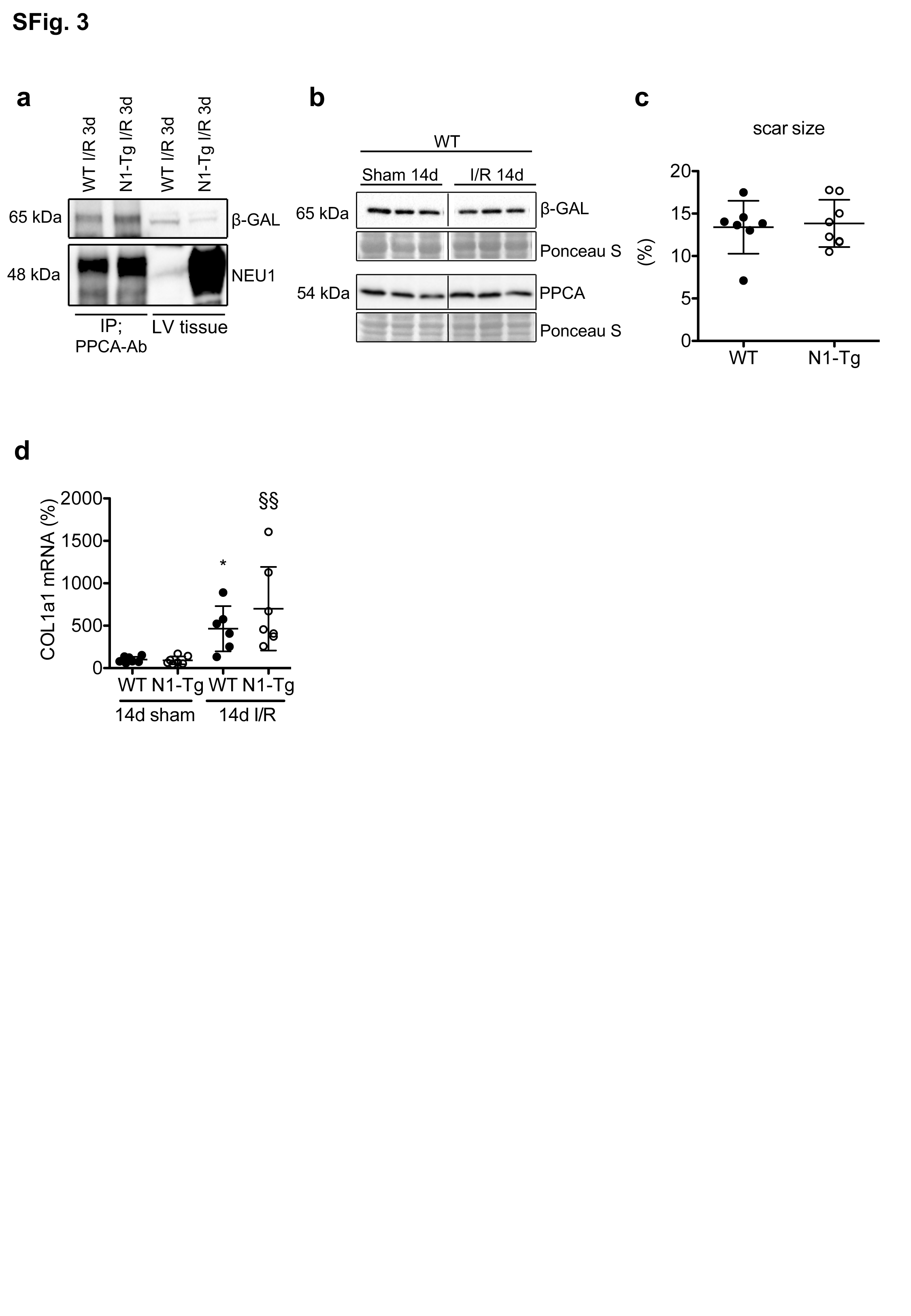

Supplement: Supplementary file 3 — Supplementary file3 (TIF 22773 kb) [file 395_2020_821_MOESM3_ESM.tif]

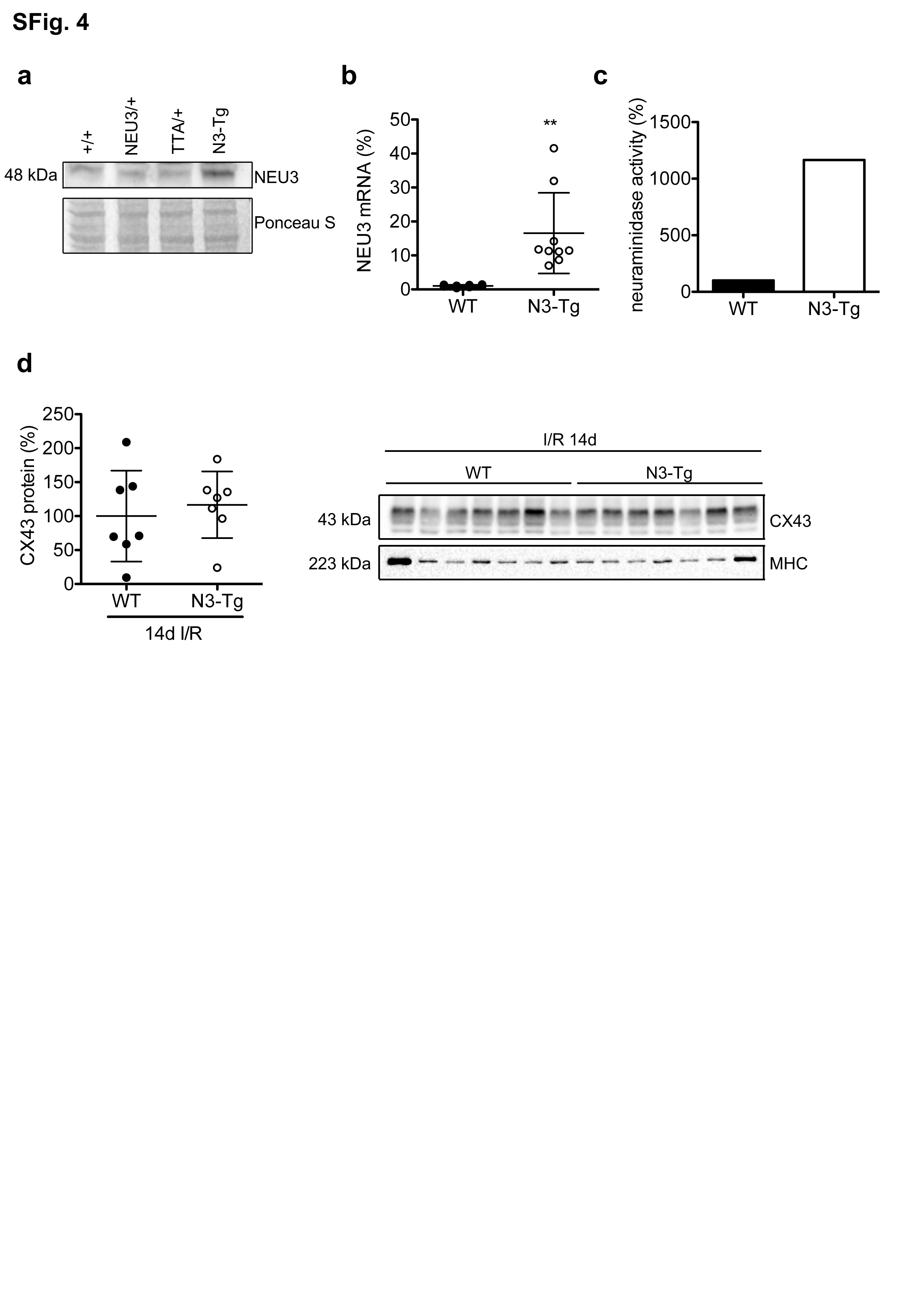

Supplement: Supplementary file 4 — Supplementary file4 (TIF 22928 kb) [file 395_2020_821_MOESM4_ESM.tif]
