# Supplementary material for: Neuraminidase-1 promotes heart failure after ischemia/reperfusion injury by affecting cardiomyocytes and invading monocytes/macrophages
Source: Basic Res Cardiol. 2020 Sep 25;115(6):62. doi: 10.1007/s00395-020-00821-z (PMC7519006; doi:10.1007/s00395-020-00821-z)
Supplement: Supplementary file 5 — Supplementary file5 (DOCX 32 kb) [file 395_2020_821_MOESM5_ESM.docx]

**Neuraminidase-1 promotes heart failure after ischemia/reperfusion injury by affecting cardiomyocytes and invading monocytes/macrophages**

Maren Heimerl^1*^, Irina Sieve^1*^, Melanie Ricke-Hoch^1^, Sergej Erschow^1^, Karin Battmer^2^, Michaela Scherr^2^ and Denise Hilfiker-Kleiner^1^

^1^Molecular Cardiology, Dept. of Cardiology and Angiology, and ^2^Dept. of Hematology, Hemostasis, Oncology and Stem Cell Transplantation, Hannover Medical School, Carl-Neuberg-Str.1, 30625 Hannover, Germany

***equally contributing first authors**

**Corresponding author**:

Denise Hilfiker-Kleiner, PhD

Dept. of Cardiology and Angiology

Hannover Medical School

Carl-Neuberg Str. 1

30625 Hannover, Germany

Phone: +49-511-532-2531, Fax: +49-511-532-3263

E-mail: hilfiker.denise@mh-hannover.de

Short title: Neuraminidase-1 promotes heart failure after ischemia/reperfusion

Category: Original Articles

Word count: 8400 words

The manuscript is not submitted elsewhere nor is under consideration for publication.

All authors have read and agreed with the submission of the manuscript.

**Acknowledgments**

The authors wish to thank Birgit Brandt, Silvia Gutzke, Lea Greune and Martina Kasten for excellent technical assistance. We thank the Core Facility for Laser Microscopy and the Research Core Unit Genomics of the Hanover Medical School.

**Funding**

This work was supported by the Deutsche Forschungsgemeinschaft (DFG) [HI 842/8-1].

Keywords: Neuraminidase 1, sialidase 1, ischemia/reperfusion, inflammation, monocytes

**Supplementary Methods**

*RNASeq and bioinformatics*

RNA Seq analysis was performed by the Research Core Unit Genomics (RCUG) of the Medical School Hanover (MHH). LV of male mice at the age of 10-12 weeks was used for analysis (n=9-10 per genotype).

Library generation, quality control, and quantification: 500 ng of total RNA per sample were utilized as input for rRNA depletion procedure with ‘NEBNext® rRNA Depletion Kit (Human/Mouse/Rat), 96 rxns’ (E6310X; New England Biolabs) followed by stranded cDNA library generation using ‘NEBNext® Ultra II Directional RNA Library Prep Kit for Illumina’ (E7760L; New England Biolabs). All steps were performed as recommended in user manualE7760 (Version 1.0_02-2017; NEB) except that all reactions were downscaled to 2/3 of initial volumes. Furthermore, one additional purification step was introduced at the end of the standard procedure, using 1.2x ‘Agencourt® AMPure® XP Beads’ (#A63881; Beckman Coulter, Inc.). cDNA libraries were barcoded by single indexing approach, using ‘NEBNext Multiplex Oligos for Illumina – Set 1’ (Index Primer 1, 2, 4, 5, 6, 7, 12). All generated cDNA libraries were amplified by 9 cycles of final PCR. Fragment length distribution of individual libraries was monitored using ‘Bioanalyzer High Sensitivity DNA Assay’ (5067-4626; Agilent Technologies). Quantification of libraries was performed by use of the ‘Qubit® dsDNA HS Assay Kit’ (Q32854; ThermoFisher Scientific). Library denaturation and Sequencing run: Equal molar amounts of seven individually barcoded libraries were pooled. Accordingly, each analyzed library constitutes 14.3% of overall flowcell capacity. The library pool was denatured with NaOH and was finally diluted to 1.5pM according to the Denature and Dilute Libraries Guide (Document # 15048776 v02; Illumina). 1.3 ml of denatured pool was loaded on an Illumina NextSeq 550 sequencer using a High Output Flowcell for 75bp single reads (#FC-404-2005; Illumina). Raw data processing and quality control: BCL files were converted to FASTQ files using bcl2fastq Conversion Software version v2.20.0.422 (Illumina). The FASTQ files were adapter and quality trimmed using Trim Galore! (version 0.4.1) with default settings as described in the User Guide except for the setting of the quality cutoff (-q/--quality) which was set to a Phred score of 15. Trim Galore! used Cutadapt (version 1.9.1) as subroutine. Quality control of FASTQ files was performed by FastQC (version 0.11.4) before and after trimming. In order to enable directional single read data import to StrandNGS V3.1.1 (see below) the reverse complements of the trimmed sequences were generated as well as the inverse quality string of each read using SeqKit (version 0.7.2) with default settings and –r (reverse sequence) and –p (complement sequence). Mapping: After trimming, FASTQ files were mapped against a reference genome with the splice-aware aligner STAR (version 2.5.0c) to generate BAM files. The BAM files were built in a 2-pass Mapping (--twopassMode Basic) and were finally sorted (--outSAMtype BAM SortedByCoordinate). All other setting have been left as default as described in the manual. The genome index files were created by STAR with default settings using Mus musculus sequence and annotation data (UCSC, build mm10) available on illumina’s iGenome site (<http://support.illumina.com/sequencing/sequencing_software/igenome.html>). The average number of reads entering the mapping process across all analyzed samples was 85.9 million. The average percentage of uniquely mapped reads was 84.6%, of reads mapped to multiple loci was 10.8%, and of unmapped reads was 4.3%. Quantification and normalization: Generated BAM files were imported to StrandNGS software (version 3.1.1) according to the following settings: Build: Mouse mm10; Transcript Annotation: Ensembl Transcripts (2013.04.03); Library layout: Directional Single End. Quantification was performed using DESeq normalization algorithm (including partial reads). The threshold of normalized read counts were set to 1. No baseline transformation was conducted.

**Supplementary Data**

**Supplementary figures**

**sFig. 1: Genotyping, protein expression and neuraminidayse activity in hNEU1 compared to WT mice**. (a) Representative gel with genotyping for the NEU1a and b alleles in a heterozygous, hNEU1 and WT mouse. (b) Representative Western blot showing NEU1 protein in the LV of WT and hNEU1 mice, Ponceau staining serves as loading control. N=2 per genotype. (c) Relative neuraminidase activity in LV tissue from hNEU1 and WT mice 3 days after sham operation after normalization to protein concentration. WT sham N=8; hNEU1 sham N=9. All values are depicted as mean ± SD. Statistical analysis was done using two-tailed t-test with Welch’s correction (c). *P<0.05 hNEU1 sham 3 d vs. WT sham 3 d

**sFig. 2: Gating strategy and representative dot plots of F4/80^+^Ly-6C^high^ (R5) and the F4/80^+^Ly-6C^low^ (R6) population in the heart and the bone marrow and isotype controls.**

(a) After collagenase digestion and filtration to remove cardiomyocytes, live cells were identified and gated by their forward and side scatter (R1). (b) The immune cells were then identified by staining with a cocktail of lineage-specific antibodies and CD11b (R2). (c) Of the Lin^-^CD11b^+^ population (R2 in panel b) the pro-inflammatory Lin^-^CD11b^+^F4/80^+^Ly-6C^high^ (R5) and the anti-inflammatory Lin^-^CD11b^+^F4/80^+^Ly-6C^low^ (R6) population was determined.

(d) Dot plot showing the isotype control from mouse heart with IgG FITC and IgG PECy7 antibodies

**sFig. 3: No difference in scar size between N1-Tg and WT mice 14 days after I/R injury.** (a) Western blot analysis of β-GAL and NEU1 protein levels after immunoprecipitation (IP) with PPCA. (b) Western blot analysis of NEU1 cofactors PPCA and β-GAL 14 days after I/R compared with sham WT LV. N=3. (c) Quantification of scar patches of N1-Tg and WT LV was done by measuring scar area in comparison to whole LV area in %. WT N = 7; N1-Tg N=8. (d) Relative COL1a1 mRNA expression in the ischemic LV of N1-Tg and WT hearts, normalized to 18S. WT sham N=8; N1Tg sham N=7; WT I/R N=6; N1-Tg I/R N=7. All values are depicted as mean ± SD. Statistical analysis was done using two-tailed t-test with Welch’s correction (c) and Two-Way-ANOVA with Bonferroni post-test (d). *P<0.05 and WT I/R 14 d vs. WT sham 14 d; §§P<0.01 N1-Tg I/R 14 d vs. N1-Tg sham 14 d

**sFig. 4: Cardiomyocyte-specific overexpression of NEU3.** (a) Representative Western blot showing NEU3 overexpression in the LV of a N3-Tg mouse as compared to WT hearts, Ponceau staining serves as loading control. (b) Relative NEU3 mRNA expression in ischemic LV of N3-Tg mice 3 days after I/R as compared with WT animals after normalization to 18S. WT N=8; N3-Tg N=9. (c) Exemplarily neuraminidase activity in ischemic LV of one N3-Tg mice compared with WT. N=1. (d) Western Blot and relative quantification, respectively, of CX43 in N3-Tg and WT LV 14 days after I/R, normalized to MHC. N=7. All values are depicted as mean ± SD. Statistical analysis was done using unpaired, two-tailed t-test with Bonferroni post-test (b, d). **P<0.01 N3-Tg I/R 3 d vs. WT I/R 3 d

**Supplementary tables**

**sTab. 1: Basal echocardiography in male hNEU1 mice.** Echocardiographic analysis was performed in 8-14 weeks old male hNEU1 and corresponding WT mice. All values are depicted as mean ± SD. Statistical analysis was done using unpaired *t*-test (FAC, LVEDA, HR) and Mann Whitney test (LVESA), respectively.

|  | **WT**  N=8 | **hNEU1**  N=9 |
| --- | --- | --- |
| FAC (%) | 62 ± 3 | 59 ± 6 |
| LVEDA (cm^2^) | 0.171 ± 0.017 | 0.177 ± 0.024 |
| LVESA (cm^2^) | 0.056 ± 0.017 | 0.073 ± 0.018 |
| HR (bpm) | 543 ± 33 | 510 ± 34 |

FAC, fractional area change; LVEDA, left ventricular enddiastolic area; LVESA, left ventricular endsystolic area; HR, heart rate (bpm, beats per minute).

**sTab. 2: Basal echocardiography in male N1-Tg mice**. Echocardiographic analysis was performed in 10-14 weeks old male N1-Tg and corresponding WT mice. All values are depicted as mean ± SD. Statistical analysis was done using unpaired *t*-test (FAC, LVEDA, HR) and Mann-Whitney test (LVESA), respectively. *P<0.05 N1-Tg vs. WT.

|  | **WT**  N=8 | **N1-Tg**  N=9 |
| --- | --- | --- |
| FAC (%) | 56 ± 5 | 53 ± 4 |
| LVEDA (cm^2^) | 0.179 ± 0.015 | 0.197 ± 0.013* |
| LVESA (cm^2^) | 0.080 ± 0.014 | 0.092 ± 0.010 |
| HR (bpm) | 550 ± 25 | 524 ± 36 |

FAC, fractional area change; LVEDA, left ventricular enddiastolic area; LVESA, left ventricular endsystolic area; HR, heart rate (bpm, beats per minute).

Primer sequences used for genotyping:

| **gene** | **Primer sequence** | **Annealing temp. (°C)** |
| --- | --- | --- |
| N1-Tg | F: 5’-TCTACGGCACGCTCTGAACCCA  R: 5’-CTCCCACACCTCCCCCTGAACC | 63 |
| TTA-Tg | F: 5’-AGCGCATTAGAGCTGCTTAATGAGGTC  R: 5’-GTCGTAATAATGGCGGCATACTATC | 65 |
| SM.129S2:  240 C 🡪 T | F: 5’-CTTAAGGGCATTGGGGTCAT  R: 5’-ATCCCTGTCCAGGAACTGGT | 60 |

Primer sequences used for real time-PCR:

| **Gene names** | **Primer sequence (5’🡪3’)** |
| --- | --- |
| *Adgre1* | For: GAGACATCCACTCTGGGCAC |
|  | Rev: GGGGCCCCTGTAGATACTGA |
|  |  |
| *ANP* | For: GCCGGTAGAAGATGAGGTCA |
|  | Rev: GGGCTCCAATCCTGTCAATC |
|  |  |
| *BCL-2* | For: AAGCTGTCACAGAGGGGCTA |
|  | Rev: CAGGCTGGAAGGAGAAGATG |
|  |  |
| *COL1a1* | ACA GAC GAA CAA CCC AAA CT |
|  | GGT TTT TGG TCA CGT TCA GT |
|  |  |
| *CX43* | For: AGGAGTTCCACCACTTTGGC |
|  | Rev: AGCGAAAGGCAGACTGTTCA |
|  |  |
| *HPRT* | For: CATTATGCCGAGGATTTGGAA |
|  | Rev: TGACATCTCGAGCAAGTCTTTCA |
|  |  |
| *NEU1* | For: GATGTTTGCCCCTGGACCTG |
|  | Rev: GTGTCCACACACAATGAGCCG |
|  |  |
| *NEU3* | For: GAAGTGCAGAGGAGGTCTTG |
|  | Rev: ATCGAAAGACACCATCACG |
|  |  |
| *TPT1* | For: TGACGAGCTGTTCTCCGACA |
|  | Rev: CGATGGCACCCTCTGTTCTA |
|  |  |
| *ZFY-1* | For: TGGAGAGCCACAAGCTAACCA |
|  | Rev: CCCAGCATGAGAAAGATTCTTC |
|  |  |
| *18S* | For: GTAACCCGTTGAACCCCATT |
|  | Rev: CCATCCAATCGGTAGTAGCG |

Antibodies used for FACS analyses:

| **Antibody** | **Conjugate** | **Supplier** |
| --- | --- | --- |
| anti-mouse CD90.2 (53-2.1), rat | PE | BD Biosciences, Erembodegem, Belgium |
| anti-mouse CD45R/B220 (RA3-6B2), rat, | PE | BD Biosciences, Erembodegem, Belgium |
| anti-mouse CD49b (DX5), rat | PE | BD Biosciences, Erembodegem, Belgium |
| anti-mouse NK1.1 (PK136), mouse | PE | BD Biosciences, Erembodegem, Belgium |
| anti-mouse Ly-6G (1A8), rat | PE | BD Biosciences, Erembodegem, Belgium |
| anti-mouse/human CD11b (M1/70), rat | APC | BioLegend, San Diego, USA |
| anti-mouse Ly-6C (AL-21), rat | PE/Cy7 | BD Biosciences, Erembodegem, Belgium |
| anti-mouse F4/80 (BM8), rat | FITC | BioLegend, San Diego, USA |
